# Fibroblast growth factor receptor family mutations as a predictive biomarker for immune checkpoint inhibitors and its correlation with tumor immune microenvironment in melanoma

**DOI:** 10.3389/fimmu.2022.1030969

**Published:** 2022-11-08

**Authors:** Wengang Zhang, Handai Xia, Rui Yang, Yuqing Zhang, Qi Zheng, Xiaoling Shang, Ni Liu, Xinchun Ma, Chenxi Wei, Hang Chen, Xin Mu, Xiuwen Wang, Yanguo Liu

**Affiliations:** ^1^ Department of Medical Oncology, Qilu Hospital of Shandong University, Jinan, Shandong, China; ^2^ School of Basic Medical Sciences, Shandong First Medical University, Jinan, China; ^3^ Department of Medical Imaging Center, Third People’s Hospital of Jinan, Jinan, China

**Keywords:** FGFR mutations, immune checkpoint inhibitors, melanoma, biomarker, tumor immune microenvironment

## Abstract

**Background:**

The emergence of immune checkpoint inhibitors (ICIs) has significantly improved the clinical outcomes of patients with metastatic melanoma. However, survival benefits are only observed in a subset of patients. The fibroblast growth factor receptor (FGFR) family genes are frequently mutated in melanoma, yet their impacts on the efficacy of ICIs remain unclear. Our study aimed to explore the association of FGFR mutations with ICIs efficacy in metastatic melanoma.

**Methods:**

The Cancer Genome Atlas (TCGA) data (PanCancer Atlas, skin cutaneous melanoma (SKCM), n = 448) in cBioPortal were collected as a TCGA cohort to investigate the association between FGFR mutations and prognosis of melanoma patients. To explore the impact of FGFR mutations on the efficacy of ICIs in melanoma, clinical and tumor whole-exome sequencing (WES) data of four ICI-treated studies from cBioPortal were consolidated as an ICIs-treated cohort. Moreover, the relationship between FGFR mutations and immunogenicity (tumor mutation burden (TMB), neo-antigen load (NAL), mismatch repair (MMR)-related genes and DNA damage repair (DDR)-related genes) of melanoma was evaluated utilizing data from the ICIs-treated cohort. The influence of FGFR mutations on the tumor immune microenvironment (TIME) of melanoma was also analyzed using the TCGA cohort.

**Results:**

In the TCGA cohort, survival in melanoma patients with or without FGFR mutations was nearly equivalent. In the ICIs-treated cohort, patients with FGFR mutations had better survival than those without (median overall survival: 60.00 vs. 31.00 months; hazard ratio: 0.58, 95% CI: 0.42-0.80; P = 0.0051). Besides, the objective response rate was higher for patients harboring FGFR mutations (55.56%) compared to wild-type patients (22.40%) (P = 0.0076). Mechanistically, it was revealed that FGFR mutations correlated with increased immunogenicity (e.g., TMB, NAL, MMR-related gene mutations and DDR-related gene mutations). Meanwhile, FGFR mutant melanoma tended to exhibit an enhanced antitumor TIME compared with its wild-type counterparts.

**Conclusions:**

Our study demonstrated that FGFR mutations is a promising biomarker in stratifying patients with advanced melanoma who might benefit from ICIs therapy.

## Introduction

Melanoma is one of the most common malignancies in skin cancer and its incidence is escalating annually (1). It is characterized by being the leading cause of skin cancer-related mortalities. Notably, the introduction of immune checkpoint inhibitors (ICIs) and targeted agents have significantly improved the survival of patients with advanced melanoma, boosting the five-year survival rate from less than 10% historically to approximately 40% currently (2–4). ICIs, especially agents targeting the cytotoxic T-lymphocyte antigen 4 (CTLA-4) and programmed death-1 (PD-1)/programmed death-ligand 1 (PD-L1), provide robust benefits for patients with advanced melanoma (5). The data from Checkmate 067 showed that the five-year survival rates for advanced melanoma patients receiving nivolumab plus ipilimumab and nivolumab were 52% and 44%, respectively (2). However, only a minority of patients respond to ICIs and benefit from them in terms of survival (6, 7). Moreover, some patients may experience substantial toxicity from ICIs in both clinical trials and real-world clinical practice (8). Therefore, identifying predictive biomarkers for ICI efficacy and elucidating the potential mechanisms modulating sensitivity to ICIs are of crucial importance.

Multiple factors have been identified to be critical in predicting or influencing the success of ICIs in the treatment of melanoma. It is acknowledged that tumor mutational burden (TMB) can predict response to ICIs across a variety of cancers, including melanoma, with higher TMB indicating a greater probability of response (9). However, some patients with low TMB respond to ICIs as well. Additionally, the optimal cutoff value of TMB has not been determined, resulting in differing perspectives on TMB among clinicians (10). It has been determined that IFN-γ (11), tumor T-cell infiltration (12) and lactate dehydrogenase (13) are associated with the efficacy of ICIs. Notwithstanding, none of them are sensitive and precise enough to identify patients who would benefit most from ICIs. Recently, it was found that gene mutations play important roles in modulating the efficacy of ICIs (14). For example, PTPRT mutant melanoma was more responsive to ICIs (15). In addition, mutations in IGF1R (16), MAP2K1/2 (17), ARID1A (18) and NOTCH4 (19) were associated with more benefit from ICIs in melanoma, with the tumor immune microenvironment (TIME) modulation and immunogenicity alteration as their potential mechanisms. Hence, it is worthwhile to identify novel key genetic mutations affecting the efficacy of ICIs and explore their potential mechanisms, thereby maximizing the therapeutic benefit of ICIs and reducing immune-related toxicities for patients with melanoma.

The fibroblast growth factor receptor (FGFR) family consist of four highly conserved transmembrane receptors, FGFR1-4, which play key roles in embryonic development, proliferation, angiogenesis, and tumor metastasis (20). There is compelling evidence that FGFR is mutated across numerous cancers and its mutation triggers the FGFR signaling pathway, hence promoting tumor progression (21). Therefore, numerous studies have been devoted to the development of agents targeting FGFR alternations to suppress cancer progression. Currently, several FGFR inhibitors, such as erdafitinib and pemigatinib, have been approved by Food and Drug Administration (FDA) for the treatment of cholangiocarcinoma with FGFR fusion or rearrangement (22, 23). In terms of metastatic urothelial carcinoma, erdafitinib, a tyrosine kinase inhibitor of FGFR1–4, showed great antitumor activity in patients with FGFR alterations (mutations or fusions) (24). RAGNAR study in 2022 ASCO showed that multiple FGFR-altered (mutations or fusions) solid tumors responded to erdafitinib. However, there are no effective targeted drugs for metastatic melanoma with FGFR mutations. Therefore, ICIs are important candidates for FGFR-mutated melanoma. What is to be noted is that studies have proved that genetic mutations can affect the efficacy of ICIs, with some mutations (IGF1R, NOTCH4 and ARID1A) favoring ICIs (16, 18, 19) whereas others (EGFR and ALK) (25, 26) weakening their efficacy. In terms of FGFR mutations, a recent study found that FGFR-altered and wild-type bladder cancers had equivalent response rates to ICIs (27). However, there is no relevant study on whether FGFR mutations influence the effectiveness of ICIs in melanoma.

In this study, survival analysis was performed using the ICI-treated melanoma cohort from cBioPortal to explore the impact of FGFR mutations on the efficacy of ICIs in melanoma. Furthermore, the immunogenicity and TIME of melanoma with and without FGFR mutations were compared to investigate the mechanisms underlying FGFR mutations in predicting the efficacy and benefit of ICIs.

## Materials and methods

### Data collection and processing

**Figure 1:**
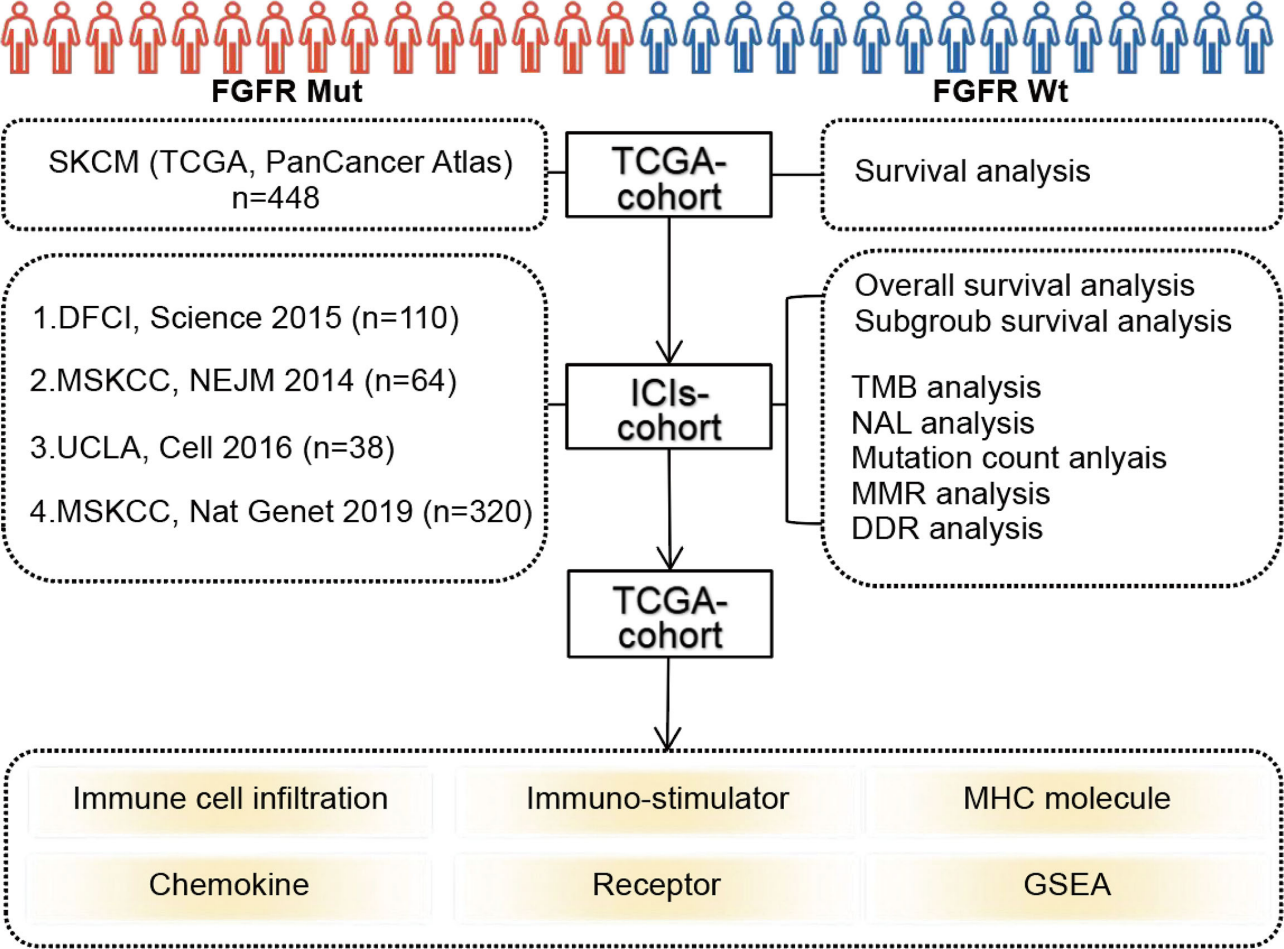
Flow diagram of the study.

The flowchart of this study was depicted in **Figure 1**. FGFR mutation frequency in pan-cancer was calculated using all TCGA PanCancer Atlas studies in cBioPortal (https://www.cbioportal.org/) (28). The TCGA data (PanCancer Atlas, SKCM, n = 448) in cBioPortal was rigorously consolidated as TCGA cohort. In addition, clinical and tumor whole-exome sequencing (WES) data concerning melanoma patients from four studies, consisting of 110 (DFCI, Science 2015) (29), 64 (MSKCC, NEJM 2014) (30), 38 (UCLA, Cell 2016) (31), and 320 (MSKCC, Nat Genet 2019) (32) samples, respectively, were downloaded to consolidated as ICIs-treated cohort. Among patients in ICIs-treated cohort, three samples with overall survival of 0 were excluded. A total of 529 samples were finally enrolled in ICIs-treated cohort. All patients in ICIs-treated cohort have been treated with ICIs, including antibodies targeting PD-(L)1 and CTLA-4. The majority of patients in the ICIs cohort were treated with ICIs in second-line or more advanced line settings. All data in TCGA cohort and ICIs-treated cohort were downloaded in the cBioPortal database (https://www.cbioportal.org/) (28). In our study, FGFR mutations (FGFR Mut) meant that melanoma patients harbor any FGFR mutations, including FGFR1, FGFR2, FGFR3 or FGFR4 mutations. In contrast, when a patient did not harbor any FGFR mutations, it was considered FGFR wild-type (FGFR Wt). All nonsynonymous mutation types, including missense, translation start site, nonstop, splice site, frameshift, and nonsense mutations, were included in this study.

### Analysis of the relationship between FGFR mutations and clinical outcomes

Firstly, survival analyses were performed based on FGFR mutation status using TCGA cohort and ICIs-treated cohort, respectively. Then, subgroup survival analyses using ICIs-treated cohort were performed based on FGFR mutation subtypes and TMB level, respectively. In the subgroup analysis based on TMB level, high TMB and low TMB were determined by the median TMB of all samples in ICIs-treated cohort. When a patient’s TMB was ≥ median TMB, it was classified as high TMB subgroup, otherwise it was considered low TMB. Response Evaluation Criteria in Solid Tumors (RECIST) version 1.1 was employed to evaluate response to ICIs. Objective response rate (ORR) reflects the percentage of patients with complete response (CR) and partial response (PR). In addition, we have constructed the nomogram for predicting survival of ICIs-treated melanoma patients by integrating clinicopathological variables including age, sex, ICIs categories, TMB, and FGFR1/2/3/4 status utilizing Sangerbox.

### Analysis of indicators relating cancer immunogenicity

Multiple parameters involving immunogenicity, including TMB, mutation count, neo-antigen load (NAL), mismatch repair (MMR)-associated gene mutations and DNA damage repair (DDR)-associated gene mutations, were compared between FGFR Mut and FGFR Wt melanoma in ICIs-treated cohort. Besides, 13 melanoma studies (DFCI, Nature Medicine 2019; UCLA, Cell 2016; Broad/Dana Farber, Nature 2012; MSKCC, Clin Cancer Res 2021; MSKCC, NEJM 2014; TCGA, Cell 2015; DFCI, Science 2015; MSKCC, JCO Precis Oncol 2017; Broad, Cell 2012; TCGA, PanCancer Atlas; Yale, Nat Genet 2012; Broad, Cancer Discov 2014; Broad Institute, Nat Genet 2015) from cBioPortal (https://www.cbioportal.org/) were used to analyze the correlation of FGFR mutation frequency with TMB (median TMB and average TMB).

### Analysis of TIME in FGFR mutant melanoma

TCGA data (PanCancer Atlas, SKCM, n = 448) from cBioPortal and RNA-seq data of corresponding samples retrieved from UCSC Xena data portal (https://xenabrowser.net) (33) were utilized to analyze the association of FGFR mutations with TIME in melanoma. CIBERSORT algorithm was used to calculate the proportion of 22 immune cells in each patient with melanoma (34). Single-sample gene set enrichment analysis (ssGSEA) method from R package GSVA was applied to calculate the infiltration level of 28 immune cell types according to the 28 published gene sets for immune cells (35, 36). ESTIMATE, a method of evaluating the fractions of stromal and immune cells, was applied to calculate stromal score (stromal content), immune score (extent of immune cell infiltration), ESTIMATE score (synthetic mark of stroma and immune) and tumor purity of each patient with melanoma (37). Four types of immune-related genes modulating TIME, including immune-stimulator related genes, major histocompatibility complex (MHC) molecule-related genes, chemokines and their receptors, were obtained from previous studies (38) and then compared between melanoma with FGFR Mut and FGFR Wt. Gene Set Enrichment Analysis (GSEA) was performed using the GSEA software (version 4.1.0) (http://www.broadinstitute.org/gsea/index.jsp) with 1000 gene-set permutations.

### Statistical analysis

The Kaplan-Meier method and the log-rank test were used to construct survival curves (overall survival (OS), disease-free survival (DSS), and progression-free survival (PFS)) and evaluate the survival analysis, respectively. Clinical parameters of continuous variables (such as TMB, mutation count and NAL) between FGFR Mut and FGFR Wt melanoma were analyzed using Mann–Whitney U test. Categorical variables (such as CR, PR, stable disease (SD), progressive disease (PD) and ORR) were compared by χ 2 test or Fisher’s exact test. Spearman correlation coefficient was calculated to evaluate the correlation of FGFR mutation frequency with median TMB or average TMB in melanoma patients. When a P value was < 0.05, it was considered statistically significant. All statistical analysis was conducted by R software (version 4.1.3), GraphPad Prism (version 9.0) or GSEA software (version 4.1.0).

## Results

### The features of FGFR mutations in melanoma

**Figure 2:**
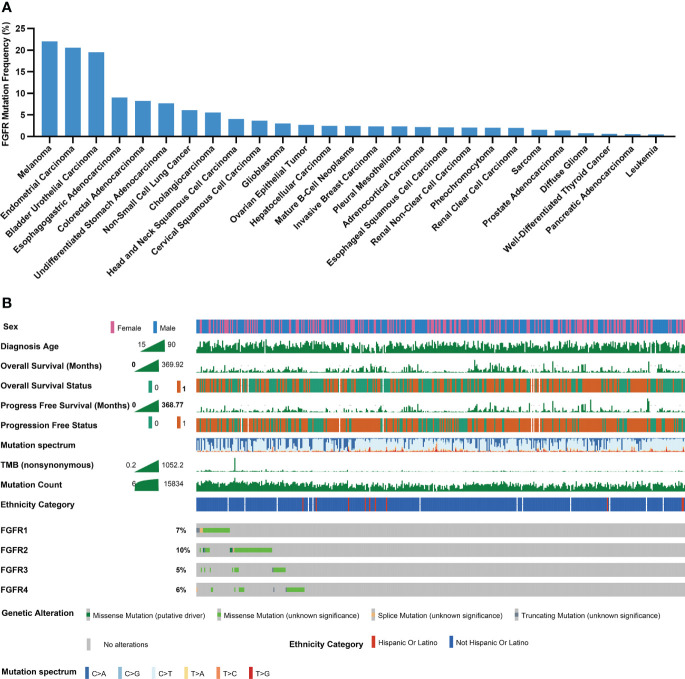
Mutational landscape of FGFR in melanoma cohorts. **(A)** The prevalence of FGFR mutations across 27 cancers. **(B)** Association of FGFR mutations and clinical characteristics in TCGA cohort (0 means no ending event occurred, 1 means ending event occurred).

Through TCGA PanCancer Atlas studies, the frequency of FGFR mutations across various cancers was evaluated. Melanoma ranked 1st with a mutation frequency of 22.05% among all 27 cancers, as depicted in **Figure 2A**, followed by endometrial carcinoma and bladder urothelial carcinoma, respectively. Subgroup analyses showed that the mutation frequencies of melanoma regarding FGFR1, FGFR2, FGFR3 and FGFR4 ranked 1st, 2nd, 2nd, and 2nd across all cancers, respectively (**Figures S1A-D**). Multiple clinical characteristics of patients in TCGA cohort, including age, gender and survival, were calculated, as shown in **Figure 2B**. In addition, FGFR mutation subtypes were counted, with P486/F/L/S, E731K, S787F and S342F being the most prevalent mutation subtypes in FGFR1, FGFR2, FGFR3, and FGFR4, respectively (**Figures S2A-D**).

### Association of FGFR mutations with survival in TCGA cohort

Patients with melanoma in TCGA cohort were mainly treated with chemotherapy and surgery. Survival analysis showed patients with FGFR Mut and FGFR Wt had comparable OS (median OS (mOS): 98.40 vs. 78.97 months, hazard ratio (HR) 0.93, 95% CI 0.67-1.30, P = 0.6520) (**Figure S3A**). Similarly, there was no significant difference in PFS (mPFS: 42.77 vs. 33.80 months, HR 0.91, 95% CI 0.69-1.19, P = 0.4938) and DSS (mDSS: 102.11 vs. 93.01 months, HR 0.84, 95% CI 0.59-1.20, P = 0.3560) between FGFR Mut and FGFR Wt patients (**Figures S3B-C**).

### Association of FGFR mutations with clinical outcomes in ICIs-treated cohort

**Figure 3:**
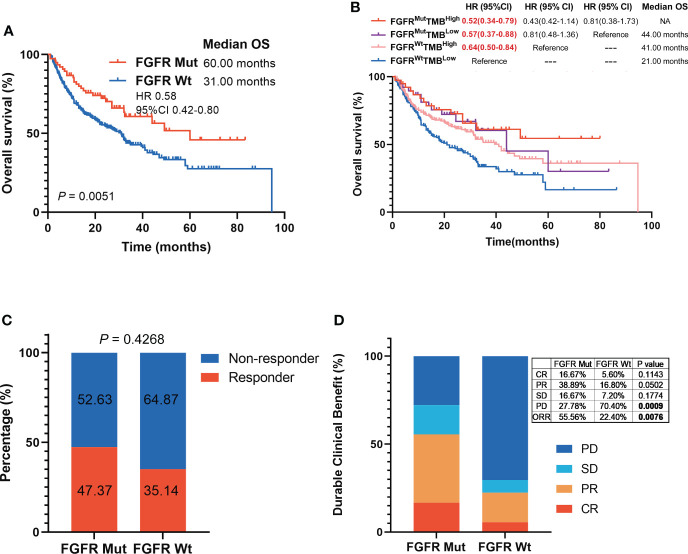
Association of FGFR mutations with melanoma clinical outcomes in ICIs-cohort. **(A)** The Kaplan-Meier survival analysis comparing OS between FGFR Mut and FGFR Wt patients in ICIs-cohort. **(B)** The Kaplan-Meier survival analyses comparing OS among FGFRMutTMBhigh, FGFRMutTMBlow, FGFRWtTMBhigh and FGFRWtTMBlow subgroups in ICIs-cohort. **(C)** Proportion of responders to ICIs in melanoma patients with FGFR mutations versus FGFR wild-type. **(D)** Comparison of the proportion of patients with complete response (CR), partial response (PR), stable disease (SD) and progression disease (PD) between FGFR Mut and FGFR Wt melanoma in ICIs-cohort.

Patients in the ICIs-treated cohort were all subjected to ICIs treatment, including anti-PD-(L)1 or anti-CTLA-4. Patient characteristics in the ICIs-treated cohort were shown in **Table 1**. Notably, patients harboring FGFR Mut had substantially longer survival with a mOS of 60.00 months compared to the FGFR Wt patients with a mOS of 31.00 months (HR 0.58, 95%CI 0.42-0.80; P = 0.0051) (**Figure 3A**). Then, ICIs-treated cohort was divided into four subgroups based on FGFR mutation status and TMB levels. The results revealed that the mOS of patients in the FGFR^Mut^TMB^high^ (HR 0.52, 95% CI 0.34-0.79, P = 0.0085), FGFR^Mut^TMB^low^ (HR 0.57, 95% CI 0.37-0.88, P = 0.0148) and FGFR^Wt^TMB^high^ (HR 0.64, 95% CI 0.50-0.84, P = 0.0057) subgroups were significantly longer than that of patients in the FGFR^Wt^TMB^low^ subgroup, respectively (**Figure 3B**). In addition, patients in the FGFR^Mut^TMB^high^ subgroup survived the longest with mOS of not reached (NR), followed by FGFR^Mut^TMB^low^ and FGFR^Wt^TMB^high^ subgroups with mOS of 44.00 months and 41.00 months, respectively, although no statistical differences were observed between them (all P > 0.05) (**Figure 3B**). FGFR mutations were correlated with responsiveness to ICIs, with higher response rate in patients with FGFR Mut (47.37%) than that in patients with FGFR Wt (35.14%) (**Figure 3C**), but the difference was not statistically significant (P = 0.4268). Meanwhile, CR rate (16.67% vs. 5.60%, P = 0.1143), PR rate (38.89% vs. 16.80%, P = 0.0502), SD rate (16.67% vs. 7.20%, P = 0.1774), and ORR (55.56% vs. 22.40%, P = 0.0076) were found to be higher in FGFR Mut group, whereas PD rate (70.40% vs. 27.78%, P = 0.0009) was higher in FGFR Wt group (**Figure 3D**).

**Table 1:** Patient characteristics in the ICIs-treated cohort.

Characteristics	No. (%)
No. of patients	529
Gender	
Male	344 (65.0)
Female	185 (35.0)
Age	
< 65	276 (52.2)
≥65	253 (47.8)
Treatment	
Anti-CTLA4	245 (46.3)
Anti-PD-(L)1	284 (53.7)
FGFR status	
FGFR Wt	77 (14.6)
FGFR Mut	452 (85.4)

### Analyses of FGFR mutation subtypes with survival in ICIs-treated cohort

**Figure 4:**
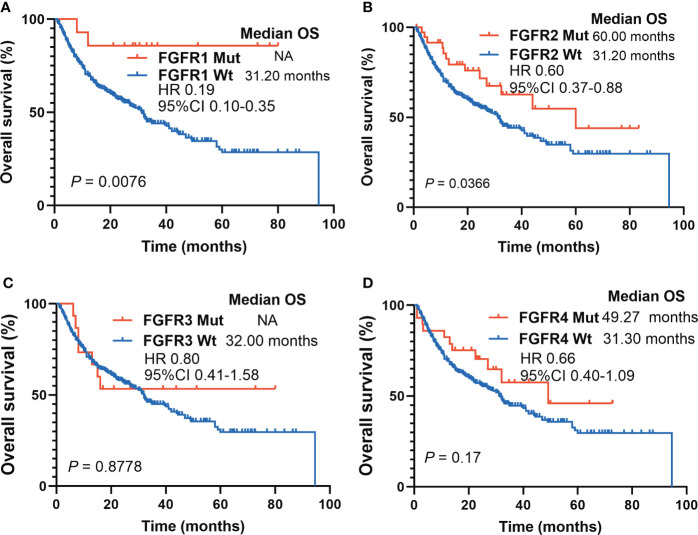
Survival analyses based on FGFR mutations subtypes in ICIs-cohort. **(A)** The Kaplan-Meier survival analysis comparing OS between FGFR1 Mut and FGFR1 Wt patients in ICIs-cohort. **(B)** The Kaplan-Meier survival analysis comparing OS between FGFR2 Mut and FGFR2 Wt patients in ICIs-cohort. **(C)** The Kaplan-Meier survival analysis comparing OS between FGFR3 Mut and FGFR3 Wt patients in ICIs-cohort. **(D)** The Kaplan-Meier survival analysis comparing OS between FGFR4 Mut and FGFR4 Wt patients in ICIs-cohort.

Subgroup survival analyses based on FGFR mutation subtypes were performed. FGFR1 Mut patients had significantly longer survival compared to patients with FGFR1 Wt (mOS: NR vs. 31.20 months; HR 0.19, 95%CI 0.10-0.35; P = 0.0076) (**Figure 4A**). Similarly, melanoma patients harboring FGFR2 Mut had a pronounced survival advantage over those with FGFR2 Wt (mOS: 60.00 months vs. 31.20 months; HR 0.60, 95%CI 0.37-0.88; P = 0.0366) (**Figure 4B**). Regarding FGFR3, a slight tendency was observed that patients harboring FGFR3 Mut benefited more from ICIs (mOS: NR vs. 32.00 months; HR 0.80, 95%CI 0.41-1.58; P = 0.8778), but no statistical difference was obtained (**Figure 4C**). Melanoma patients harboring FGFR4 Mut showed a similar tendency, whose survival was longer than those with FGFR4 Wt, though the difference was not statistically significant (mOS: 49.27 months vs. 31.30 months; HR 0.66, 95%CI 0.40-1.09; P = 0.17) (**Figure 4D**).

### Construction of the nomogram to predict survival of melanoma patients

**Figure 5:**
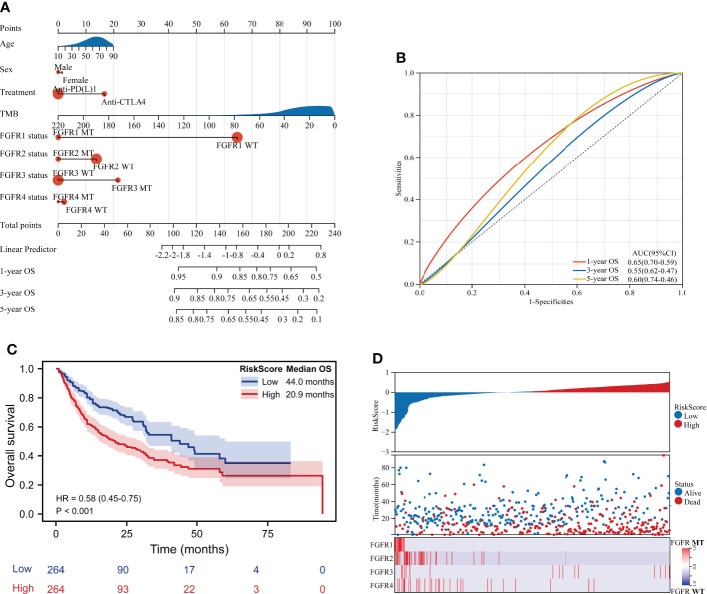
Construction of the nomogram to predict survival of melanoma patients. **(A)** A nomogram integrating clinicopathological variables including age, sex, ICIs categories, TMB, and FGFR1/2/3/4 status to predict the 1-year OS, 3-year OS and 5-year OS of patients in ICIs-treated cohort. **(B)** The ROC curve showed the predictive performance of the nomogram. **(C)** Survival curve of OS for patients from the ICIs-treated cohort based on risk score. **(D)** The risk score map exhibited the risk score level, survival status and FGFR status for each patient from the ICIs-treated cohort.

A nomogram, integrating clinicopathological variables including age, sex, ICIs categories, TMB, and FGFR1/2/3/4 status, was formulated to predict the 1-year OS, 3-year OS and 5-year OS of those ICIs-treated melanoma patients based on multivariable analysis (**Figure 5A**). As shown in **Figure 5B**, the receiver operating characteristic (ROC) curve showed that nomogram had relatively stronger predictability for 1-year OS, 3-year OS, and 5-year OS, with area under curves (AUC) of 0.65 (95% CI 0.59-0.70), 0.55 (95% CI 0.47-0.62) and 0.60 (95% CI 0.46-0.74), respectively. (**Figure 5B**). Besides, we have calculated the risk score of each patient based on multivariable analysis. Consequently, patients in the low-risk score group had significantly longer survival than those in the high-risk score group (mOS: 44.0 months vs. 20.9 months; HR 0.58, 95%CI 0.45-0.75; P < 0.001) (**Figure 5C**). The risk map exhibited that patients in in the low-risk score group had lower incidence of dead events and higher incidence of FGFR mutation (**Figure 5D**).

### Association of FGFR mutations with parameters involving immunogenicity

**Figure 6:**
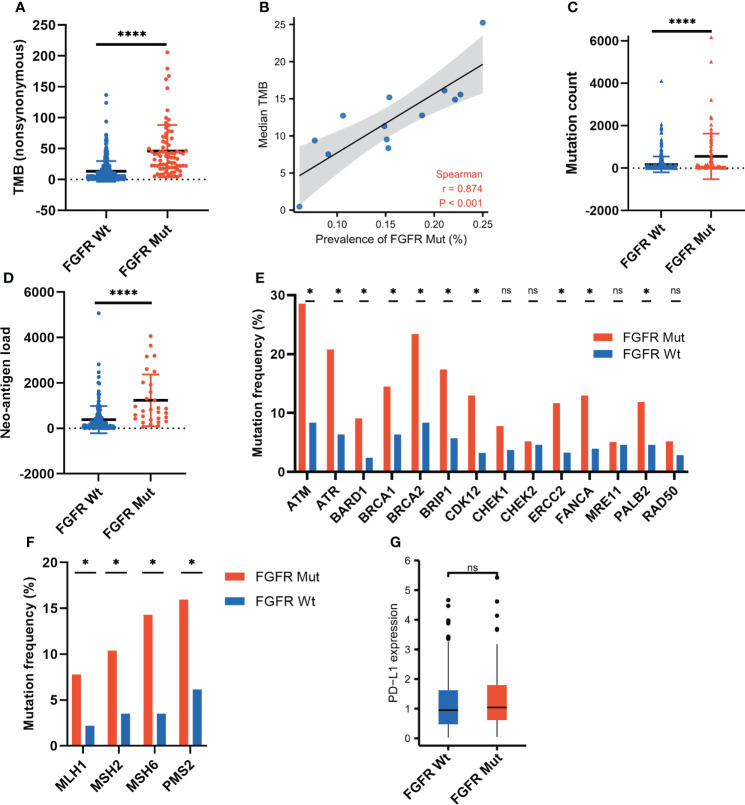
Association of FGFR mutations with parameters involving immunogenicity. **(A)** Comparison of TMB between FGFR Mut and FGFR Wt melanoma in ICIs-cohort. **(B)** Correlation of FGFR mutation frequency with median TMB in 13 melanoma studies. **(C)** Comparison of mutation count between FGFR Mut and FGFR Wt melanoma in ICIs-cohort. **(D)** Comparison of neo-antigen burden between FGFR Mut and FGFR Wt melanoma in ICIs-cohort. **(E)** Comparison of DDR-related gene mutations between FGFR Mut and FGFR Wt melanoma in ICIs-cohort. **(F)** Comparison of MMR-related gene mutations between FGFR Mut and FGFR Wt melanoma in ICIs-cohort. **(G)** Comparison of PD-L1 expression between FGFR Mut and FGFR Wt melanoma using TCGA data (ns = not significant, *P < 0.05, ****P < 0.0001).

To explore the underlying mechanisms of FGFR mutation affecting ICIs efficacy, various immunogenicity-related parameters were analyzed. FGFR mutation was associated with higher TMB (P < 0.0001), as shown in **Figure 6A**. Furthermore, 13 melanoma cohorts were employed to analyze the correlation between FGFR mutation frequency and TMB. A strongly positive correlation was found between FGFR mutation frequency and median TMB (r = 0.874, P < 0.001) (**Figure 6B**) or average TMB (P < 0.001) (**Figure S4**). Besides, we observed higher mutation count in FGFR Mut melanoma compared to its Wt counterparts (P < 0.0001) (**Figure 6C**). Likewise, compared with wild-type melanoma, FGFR Mut melanoma exhibited higher NAL (P < 0.0001) (**Figure 6D**). Given the close association of DDR or MMR process with tumor immunogenicity, the mutation frequencies of nine DDR genes and four MMR genes were examined. Higher mutation frequencies of DDR genes (ATM, ATR, BARD1, BRCA1, BRCA2, CDK12, ERCC2, FANCA and PALB2) were detected in FGFR Mut melanoma (all P < 0.05) (**Figure 6E**). Consistently, four MMR genes, including MLH1, MSH2, MSH6 and PMS2, mutated more frequently in FGFR Mut melanoma (all P < 0.05) (**Figure 6F**). In terms of PD-L1, there was a tendency that FGFR Mut melanoma expressed higher levels of PD-L1 than Wt melanoma (P > 0.05) (**Figure 6G**).

### Association of FGFR mutations with immune cell infiltration in the TIME

**Figure 7:**
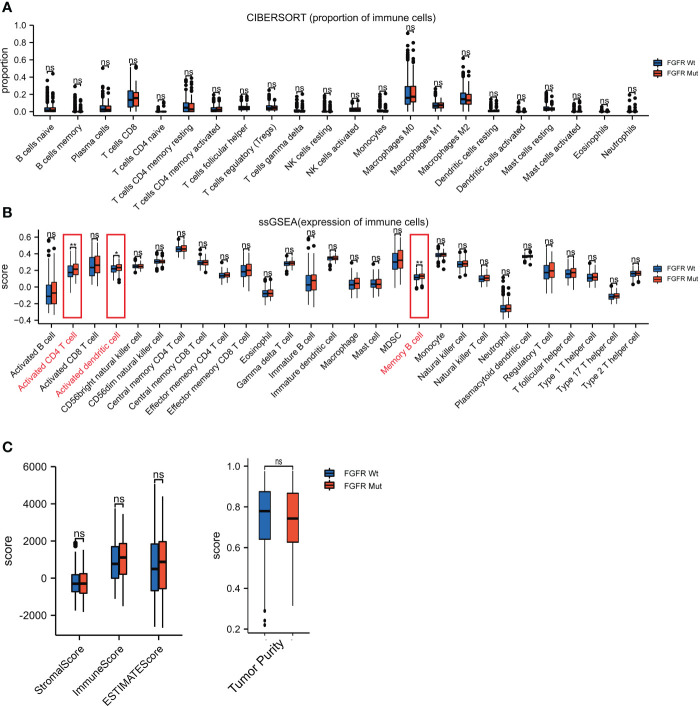
Difference of immune cell infiltration between FGFR Mut and FGFR Wt melanoma. **(A)** Comparison of proportion of immune cells between FGFR Mut and FGFR Wt melanoma. **(B)** Comparison of expression of immune cells between FGFR Mut and FGFR Wt melanoma. **(C)** Comparison of immune-related score between FGFR Mut and FGFR Wt melanoma (ns = not significant, *P < 0.05, **P < 0.01).

CIBERSORT, ssGSEA and ESTIMATE were utilized to assess the impact of FGFR mutations on the TIME of melanoma. As shown in **Figure 7A**, the FGFR Mut melanomas exhibited a mild tendency of higher proportion of anti-tumor immune cells, such as CD8+ T cells, activated CD4+ memory T cells, activated DC, activated NK and M1 macrophages, but the difference was not statistically significant (all P > 0.05). In contrast, lower proportion of M2 macrophages was observed in FGFR Mut melanoma (P > 0.05) (**Figure 7A**). Notably, ssGSEA exhibited that activated CD4+ T cells, activated DC and memory B cells were significantly abundant in FGFR Mut melanoma (all P < 0.05) (**Figure 7B**). Furthermore, immune cell infiltration levels were evaluated based on FGFR mutant subtypes and similar results were obtained (**Figures S5, S6**). Immune score and ESTIMATE score, calculated by ESTIMATE, were higher in FGFR Mut melanoma (**Figure 7C**), but not statistically significant, which were in line with results from CIBERSORT. According to these findings, we tentatively concluded that FGFR Mut melanomas were associated with increased infiltration of immune cells, which are essential mediators of ICIs to kill tumor cells.

### Association of FGFR mutations with the TIME signatures

**Figure 8:**
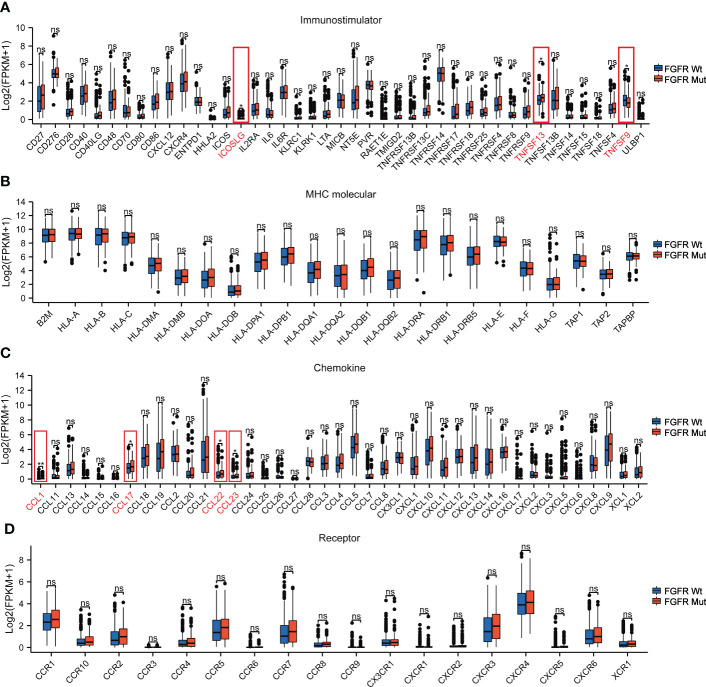
The association of FGFR Mut with anti-tumor immunity signatures in melanoma. **(A)** The expression levels of immuno-stimulator related genes in FGFR Mut versus FGFR Wt melanoma. **(B)** The expression levels of MHC molecule related genes in FGFR Mut versus FGFR Wt melanoma. **(C, D)** Comparison of chemokines and their receptors between FGFR Mut and FGFR Wt melanoma (ns = not significant, *P < 0.05, **P < 0.01).

To further elucidate the impact of FGFR mutations on the TIME of melanoma, four types of pivotal signatures modulating TIME were analyzed. Firstly, we compared the expression levels of 43 immune-stimulator genes between FGFR Mut and FGFR Wt melanoma, with the majority of genes expressing higher in FGFR Mut melanoma, especially ICOSLG and TNFSF13 (P < 0.05) (**Figure 8A**). Secondly, diverse MHC molecules were evaluated and it was discovered that they were expressed slightly higher in FGFR Mut melanoma, though the difference was not statistically significant (all P > 0.05) (**Figure 8B**). Thirdly, chemokines and their receptors were explored. Regarding chemokines, CCL1, CCL17, CCL22 and CCL23, were significantly increased in FGFR Mut melanoma (all P < 0.05). For other chemokines, most of them tended to express higher in FGFR Mut melanoma, such as CCL5, CCL19, CXCL9, CXCL10, CXCL11, CXCL13 and CXCL14 (all P > 0.05) (**Figure 8C**). Regarding chemokine receptors, FGFR Mut melanoma expressed higher levels of CCR5, CCR7, CXCR3, CXCR4 and CXCR6 than their wild-type counterparts, but no statistical difference was observed (all P > 0.05) (**Figure 8D**). Furthermore, subgroup analyses were performed based on FGFR mutation subtypes, and the results showed that the expression levels of immune-stimulators, MHC, chemokines and their receptors in melanoma with FGFR mutation subtypes were mostly similar with the results above (**Figures S7-S10**). Meanwhile, immune-related signatures (including immune cell infiltration levels, immuno-stimulators, MHC and chemokines) were compared between patients with FGFR^Mut^TMB^high^ and those with FGFR^Wt^TMB^low^. Consequently, there was a mild tendency for most immune-related signatures were more highly expressed in melanomas with FGFR^Mut^TMB^high^, but most did not show a statistically significant difference (**Figure S11**).

### GSEA analysis

**Figure 9:**
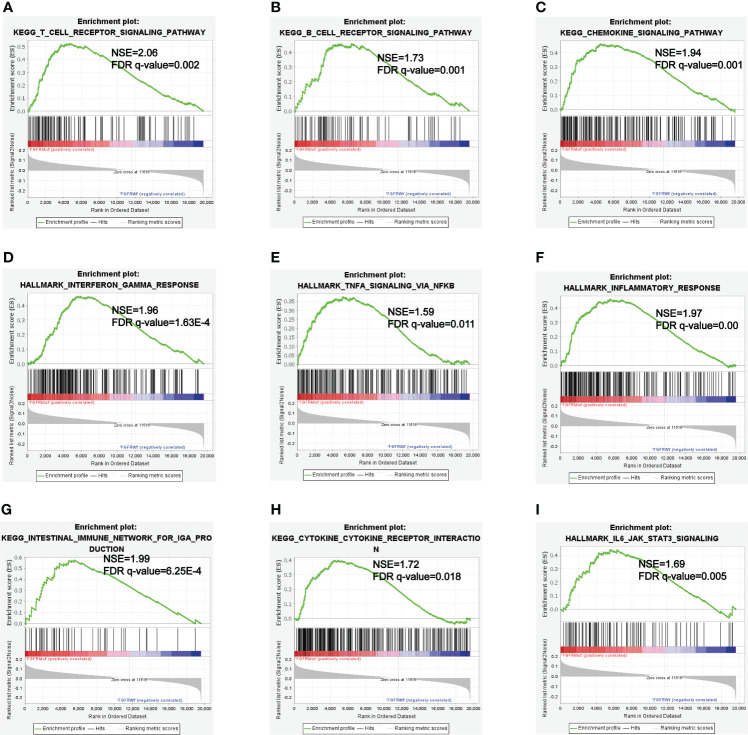
**(A-I)** Enriched Gene Sets in FGFR Mut and FGFR Wt melanoma.

GSEA analyses were performed to further explore the potential pathways by which FGFR mutations modulated the efficacy of ICIs. KEGG_T_CELL_RECEPTOR and KEGG_B_CELL_RECEPTOR signaling pathways were enriched in FGFR Mut melanoma, both of which played vital roles in modulating immune surveillance of B cells and T cells (**Figures 9A, B**). Besides, evident enrichment of anti-tumor immunity-related signatures in FGFR Mut melanoma was observed (such as KEGG_CHEMOKINE, INTERFERON_GAMMA_RESPONSE, TNFA_SIGNALING, INFLAMMATORY_ RESPONSE) (**Figures 9C-H**). IL6_JAK_STAT3_SIGNALING, a typical pathway related to TIME regulation, was found to be abundant in FGFR Mut melanoma (**Figure 9I**).

## Discussion

ICIs have significantly prolonged the survival of patients with metastatic melanoma (2). Nevertheless, only a subset of patients could benefit from ICIs (6). Indeed, the factors that influence ICI efficacy are extremely diverse and complex. Besides the widely recognized TMB, an increasing number of studies have demonstrated that gene mutations exert considerable impacts on the efficacy of ICIs, with some mutations favoring ICIs and some attenuating ICIs (14, 16, 25, 26). Notably, FGFR family driver genes are frequently mutated in melanoma, whereas its influence on ICIs efficacy in melanoma remains unknown. In this study, we found that melanoma patients with FGFR mutations who were treated with ICIs apparently survived longer than those with FGFR wild-type. Besides, increased immunogenicity and enhanced anti-tumor immunity in FGFR mutant melanoma could be the potential mechanisms that contribute to melanoma with FGFR mutations being more responsive to ICIs.

FGFR, a subfamily of receptor tyrosine kinases, comprises four members of FGFR1-4 (20). Similar to EGFR, FGFR is driver gene playing key roles the development of cancer (21). Aberrant FGFR could induce proliferation and migration of cancer cells (21). However, the survival of FGFR mutant melanoma was equivalent to that of the wild-type in our study. Substantial studies have proven that some driver gene mutations play crucial roles in modulating the efficacy of ICIs. Representatively, lung cancer harboring EGFR mutations is generally not considered for treatment with ICIs given that multiple clinical trials have found that ICIs provide limited survival benefit for this particular population (26), whereas KRAS mutation is a favorable biomarker for ICIs benefit (39). In terms of FGFR, it was found that ICIs provided comparable survival benefit for metastatic urothelial cancer with and without FGFR3 mutation (27). Strikingly, we found that melanoma with FGFR mutations benefited more from ICIs than their wild-type counterparts. Subgroup analysis based on FGFR mutation subtypes discovered similar results, especially for FGFR1 and FGFR2. Therefore, not only should melanoma patients with FGFR mutations be considered for treatment with ICIs, but they should also be given priority access. With the distinct survival benefit from ICIs in patients with FGFR mutations compared to these wild-type, FGFR mutations could be a novel biomarker for stratifying a dominant subgroup of patients with advanced melanoma for ICIs therapy.

It is well-accepted that immunogenicity plays a critical role in the activation of anti-tumor immune cells to enhance ICIs efficacy (40). Increased TMB is associated with the generation of neoantigens, representing enhanced immunogenicity (41). In the study, we found that FGFR mutant melanoma exhibiting increased TMB, which could be an important factor behind their more survival benefit from ICIs. Likewise, higher NAL was identified in FGFR mutant melanoma, providing greater evidence for prolonged survival and high response rate of FGFR mutant melanoma patients who received ICIs. Besides, the mutation frequencies of MMR-related genes and DDR-related genes were higher in FGFR mutant melanoma, correlating with genomic instability (42, 43), thereby promoting the effectiveness of ICIs in killing cancer cells. Collectively, we speculated that FGFR mutations would enhance the immunogenicity of melanoma, thereby favoring ICIs efficacy.

It is well acknowledged that the cancer-immunity cycle plays key roles in recognizing and eliminating cancer cells, which is an indispensable process in ICIs promoting anti-tumor immune response (44). Of note, multiple factors are involved in modulating the process of the cancer-immunity cycle. The presentation of cancer antigens by antigen presenting cells is the crucial first step (45). Dendritic cell, the most potent antigen-presenting cells (46), infiltrated more pronounced in FGFR mutant melanoma, which could intensify antigen presentation and T cell activation. Next, trafficking of the activated effector T cells into tumors guarantees its function (44). Higher expression of chemokines (e.g., CCL17 and CCL22) that attract anti-tumor immune cells (47–49) was found in FGFR mutant melanoma. Consistently, immune cells, such as activated CD4+ T cell and memory B cell, were more abundant in FGFR mutant melanoma. Then, specific recognition *via* the interaction between T cell receptor (TCR) on T cell and MHC on tumor cell is the important final step ensuring the cancer-immunity cycle (44). Thus, the mild tendency of increased MHC expression in FGFR mutant melanoma (all P values > 0.05) could improve the efficacy of ICIs. Meanwhile, significant enrichment of B_CELL_RECEPTOR and T_CELL_RECEPTOR pathways in FGFR mutant melanoma may further reinforce this crucial last step. Collectively, FGFR mutations predominantly boost essential processes of the cancer-immunity cycle in melanoma, which partially explains why patients with FGFR mutations benefited more from ICIs.

Inflammatory TIME is universally acknowledged to be associated with high response to ICIs (50, 51). GSEA showed that patients with FGFR mutations were more abundant in immunoinflammatory-related hallmark, such as INTERFERON_GAMMA_RESPONSE, INFLAMMATORY_RESPONSE and KEGG_CHEMOKINE (52). Therefore, it is speculated that FGFR mutations can facilitate the formation of an inflammatory TIME, which synergistically promotes ICIs to activate immune cells to kill cancer cells.

In this study, we comprehensively investigated the influence of FGFR mutations on the efficacy of ICIs in melanoma. Meanwhile, a nomogram for predicting survival of melanoma patients treated with ICIs was constructed. Furthermore, the association of FGFR mutations with immunogenicity, factors modulating the cancer-immunity cycle, and pathway enrichment were investigated to unravel the potential mechanisms of FGFR mutations affecting ICIs efficacy. Notwithstanding, there are certain limitations for this study. Firstly, the sample size of ICIs-treated cohort from public database was relatively small, especially in terms of the number of FGFR mutant patients. Therefore, prospective research with larger sample size is required for further verification. Secondly, the potential associations of FGFR mutations with immunogenicity and the cancer-immunity cycle were explored exclusively based on the analysis of public databases, which could influence the reliability of the findings. Therefore, biological validation by *in vitro* and *in vivo* experimentation is necessary. Thirdly, a certain degree of study heterogeneity existed. Patients in the ICIs-treated cohort were treated with different ICIs, including anti-PD-(L)1 and anti-CTLA-4. FGFR mutations may affect the efficacy of different ICIs differently. Fourthly, the data in this study were extracted from public database and some specific information is not available, which can impair the reliability of our results. Therefore, the predictive value of FGFR mutations in specific ICIs warrants further investigation.

## Conclusions

In the study, we first demonstrated that melanoma patients with FGFR mutations benefited more from ICIs compared with their wild-type counterparts. FGFR mutation subtypes (FGFR1, FGFR2, FGFR3 and FGFR4) showed similar results. In conclusion, it was revealed that FGFR mutations could be a favorable biomarker in predicting the efficiency of ICIs for melanoma. Mechanistically, FGFR mutations were strongly associated with strengthened tumor immunogenicity and inflamed antitumor immunity, which could be the underlying mechanisms for FGFR-mutated melanomas benefit more from ICIs.

## Data availability statement

The original contributions presented in the study are included in the article/**Supplementary Material**. Further inquiries can be directed to the corresponding authors.

## Author contributions

Conceptualization: YL and XW; methodology: WZ and RY; software: WZ, XS, and YZ; validation: QZ and HX; formal analysis: NL; XCM and XM; resources: CW; data curation: HC; writing—original draft preparation: WZ; writing—review and editing: YL; visualization: WZ; supervision: XW; project administration: XW; funding acquisition: XW and YL. All authors contributed to the article and approved the submitted version.

## Funding

This study was funded by grants from the National Natural Science Foundation of China (No. 81874044) and the Shandong Provincial Natural Science Foundation (No. ZR2020MH236 and No. ZR2019MH050).

## Conflict of interest

The authors declare that the research was conducted in the absence of any commercial or financial relationships that could be construed as a potential conflict of interest.

## Publisher’s note

All claims expressed in this article are solely those of the authors and do not necessarily represent those of their affiliated organizations, or those of the publisher, the editors and the reviewers. Any product that may be evaluated in this article, or claim that may be made by its manufacturer, is not guaranteed or endorsed by the publisher.

## Abbreviations

CR, Complete response; CTLA-4, cytotoxic T-lymphocyte antigen 4; DSS, disease-free survival; DDR, DNA damage repair; FGFR Mut, FGFR mutations; FGFR Wt, FGFR wild-type; FGFR, fibroblast growth factor receptor; FDA, Food and Drug Administration; GSEA, Gene Set Enrichment Analysis; HR, hazard ratio; ICIs, immune checkpoint inhibitors; MHC, major histocompatibility complex; MMR, mismatch repair; NAL, neo-antigen load; NA, not attached; ORR, objective response rate; OS, overall survival; PR, partial response; PD-1, programmed death-1; PD-L1, programmed death-ligand 1; PFS, progression-free survival; PD, progressive disease; RTKs, receptor tyrosine kinases; ssGSEA, single-sample gene set enrichment analysis; SKCM, skin cutaneous melanoma; SD, stable disease; TCR, T cell receptor; TCGA, The Cancer Genome Atlas; TIME, tumor immune microenvironment; TMB, tumor mutation burden; WES, whole-exome sequencing.
