# Supplementary material for: Fibroblast growth factor receptor family mutations as a predictive biomarker for immune checkpoint inhibitors and its correlation with tumor immune microenvironment in melanoma
Source: Front Immunol. 2022 Nov 8;13:1030969. doi: 10.3389/fimmu.2022.1030969 (PMC9681151; doi:10.3389/fimmu.2022.1030969)
Supplement: Supplementary file 1 [file DataSheet_1.docx]

Supplementary material


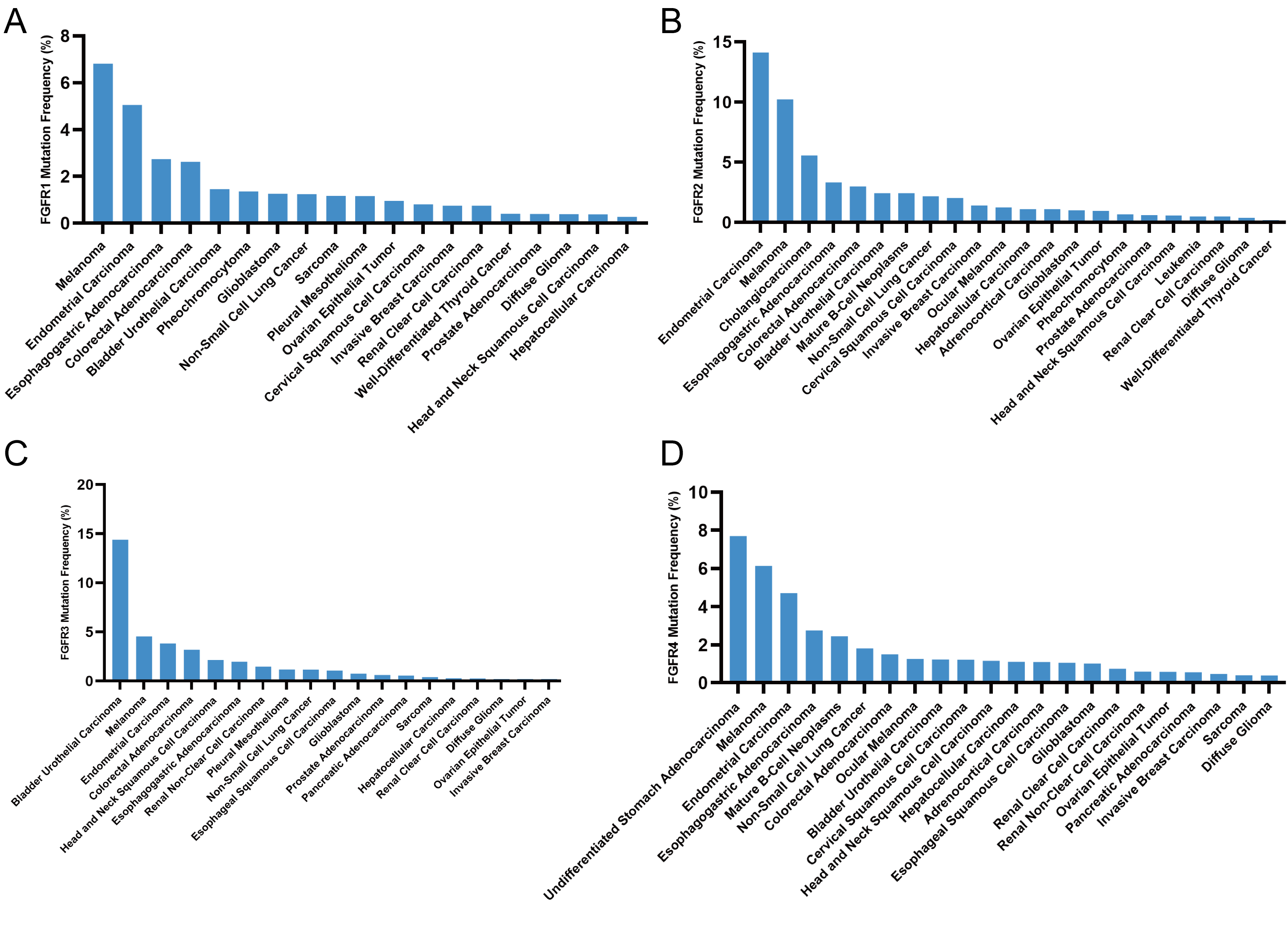


**FIGURE S1|** The mutation frequencies of FGFR subtype in melanoma. (**A**) FGFR1 mutation frequency. (**B**) FGFR2 mutation frequency. (**C**) FGFR3 mutation frequency. (**D**) FGFR4 mutation frequency.


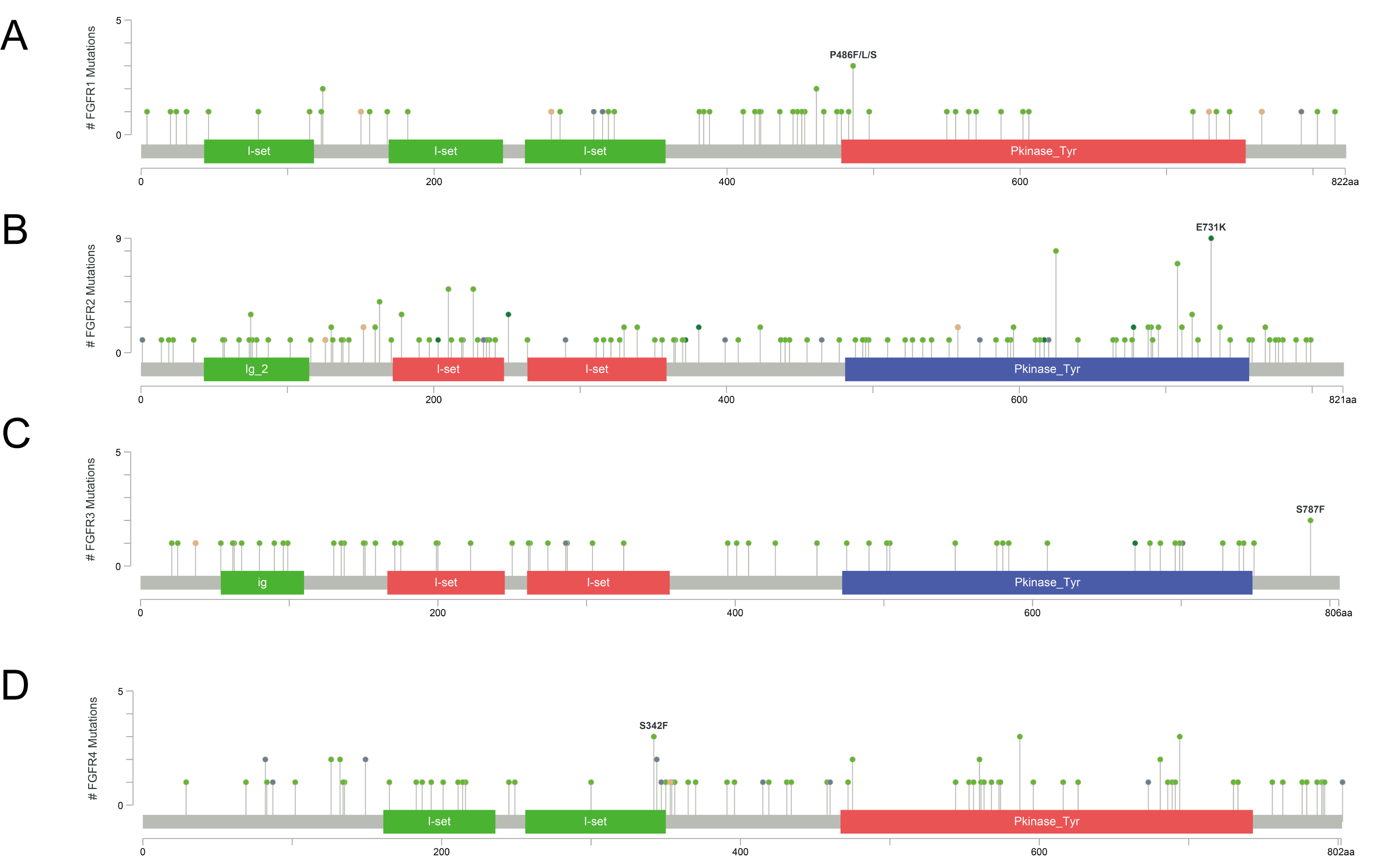


**FIGURE S2|** Lollipop plot showed the distribution of various FGFR mutation subtypes in melanoma.


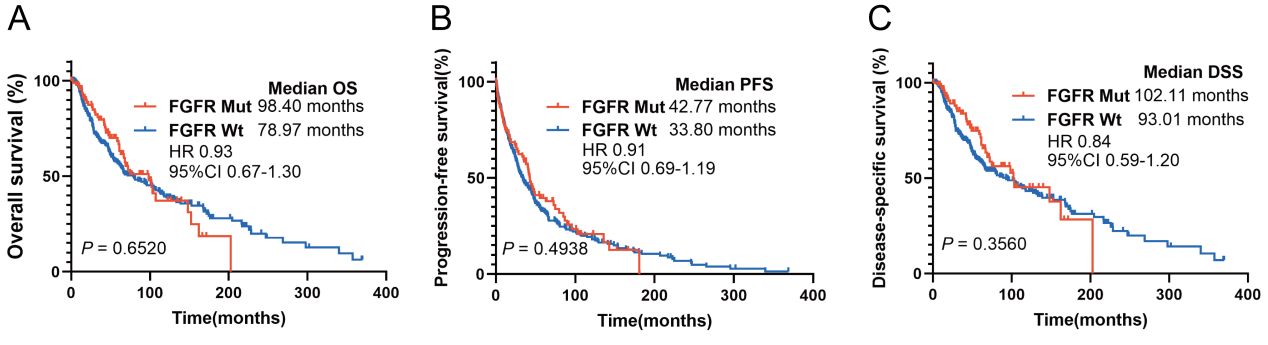


**FIGURE S3|** Association of FGFR mutations with melanoma survival in TCGA cohort. (**A**) The Kaplan-Meier survival analysis comparing OS between FGFR Mut and FGFR Wt patients in TCGA cohort. (**B**) The Kaplan-Meier survival analysis comparing PFS between FGFR Mut and FGFR Wt patients in TCGA cohort. (**C**) The Kaplan-Meier survival analysis comparing DSS between FGFR Mut and FGFR Wt patients in TCGA cohort.


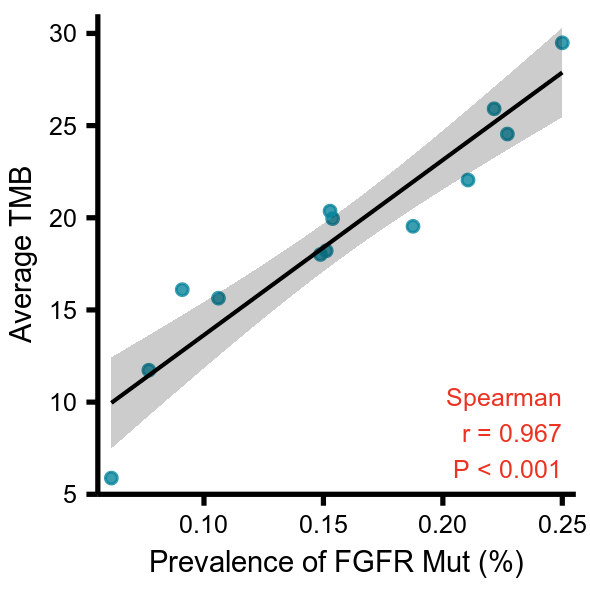


**FIGURE S4|** Correlation of FGFR mutation frequency with average TMB in 13 melanoma studies.


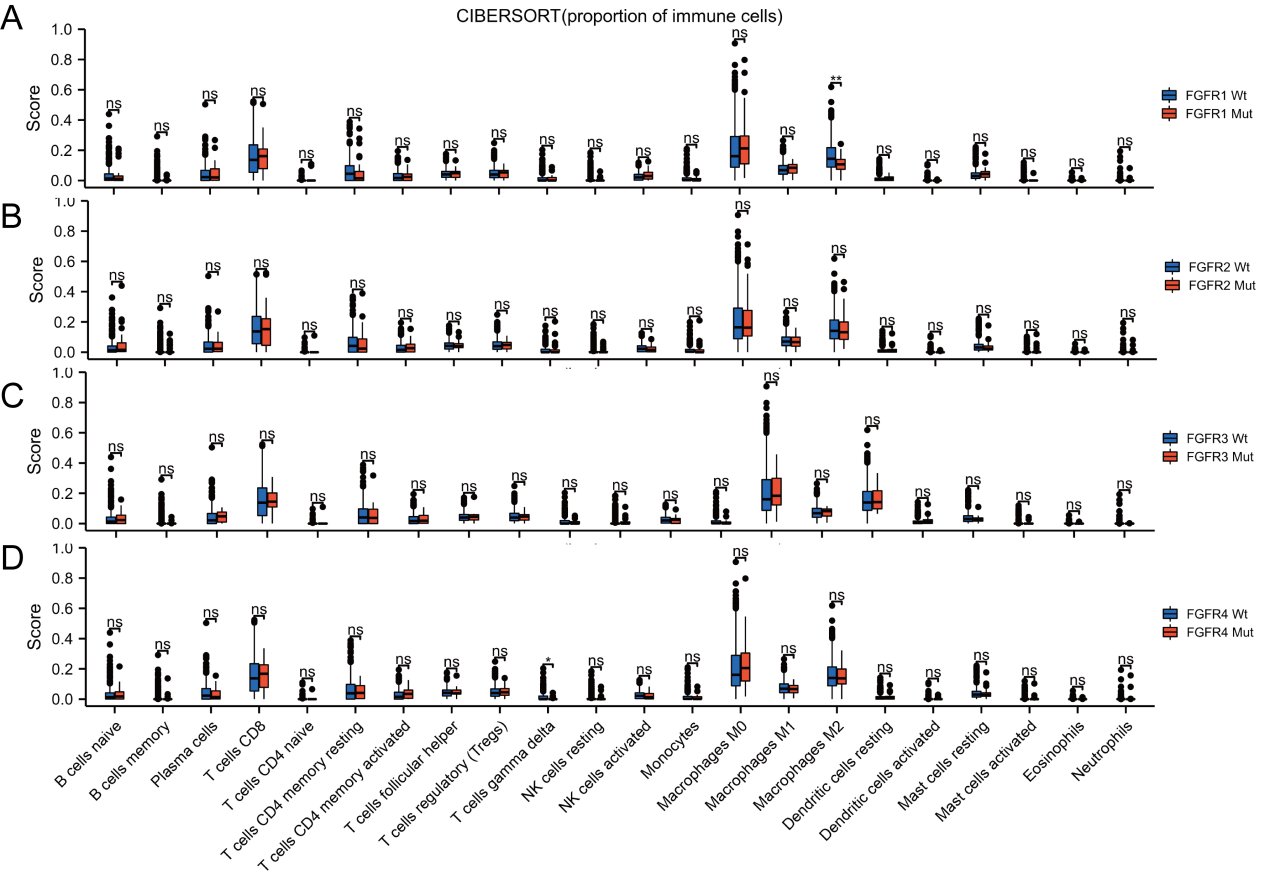


**FIGURE S5|** Comparison of proportion of immune cells in melanoma harboring FGFR mutation subtypes with their wild-type counterparts.


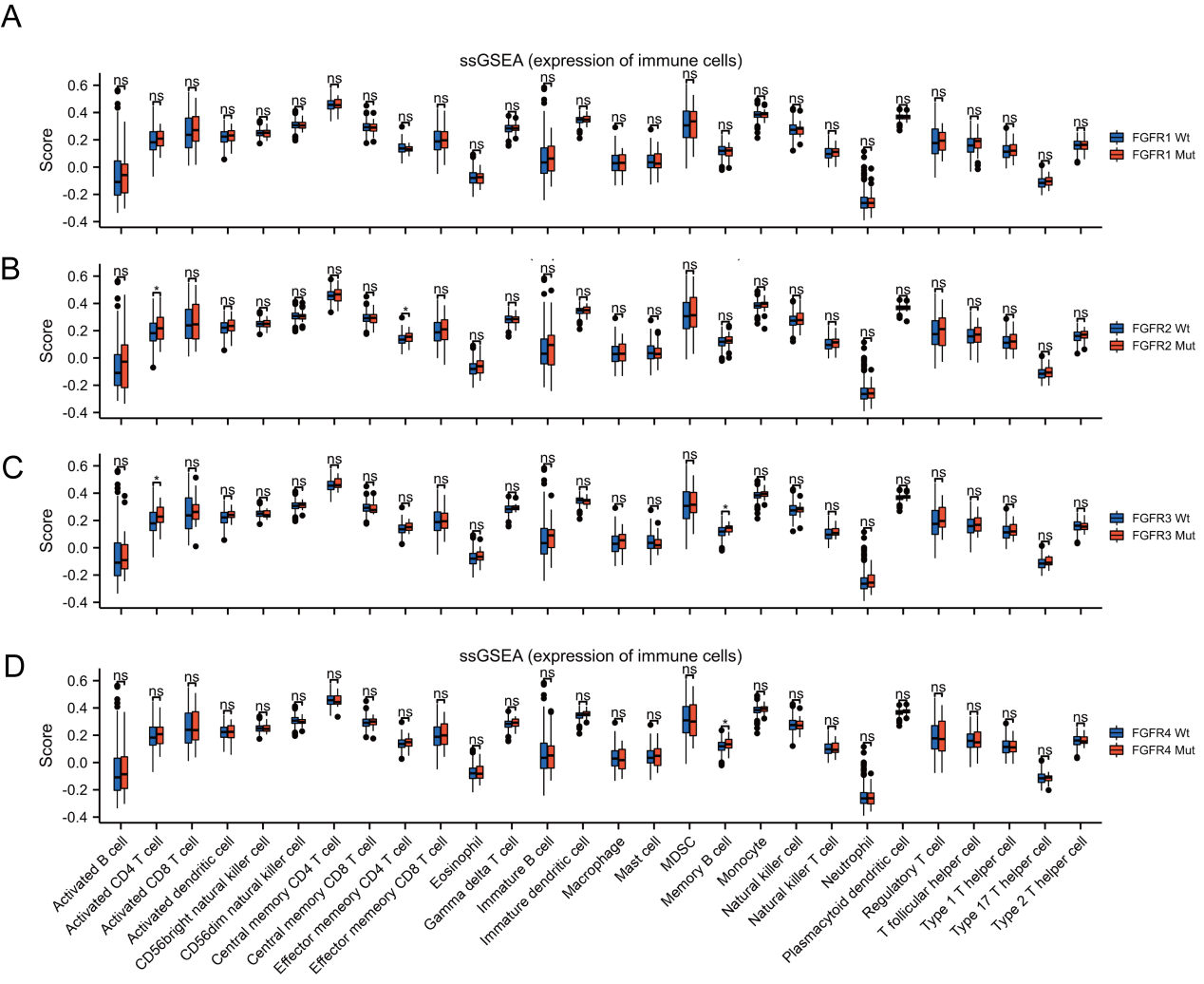


**FIGURE S6|** Comparison of expression of immune cells in melanoma harboring FGFR mutation subtypes with their wild-type counterparts.


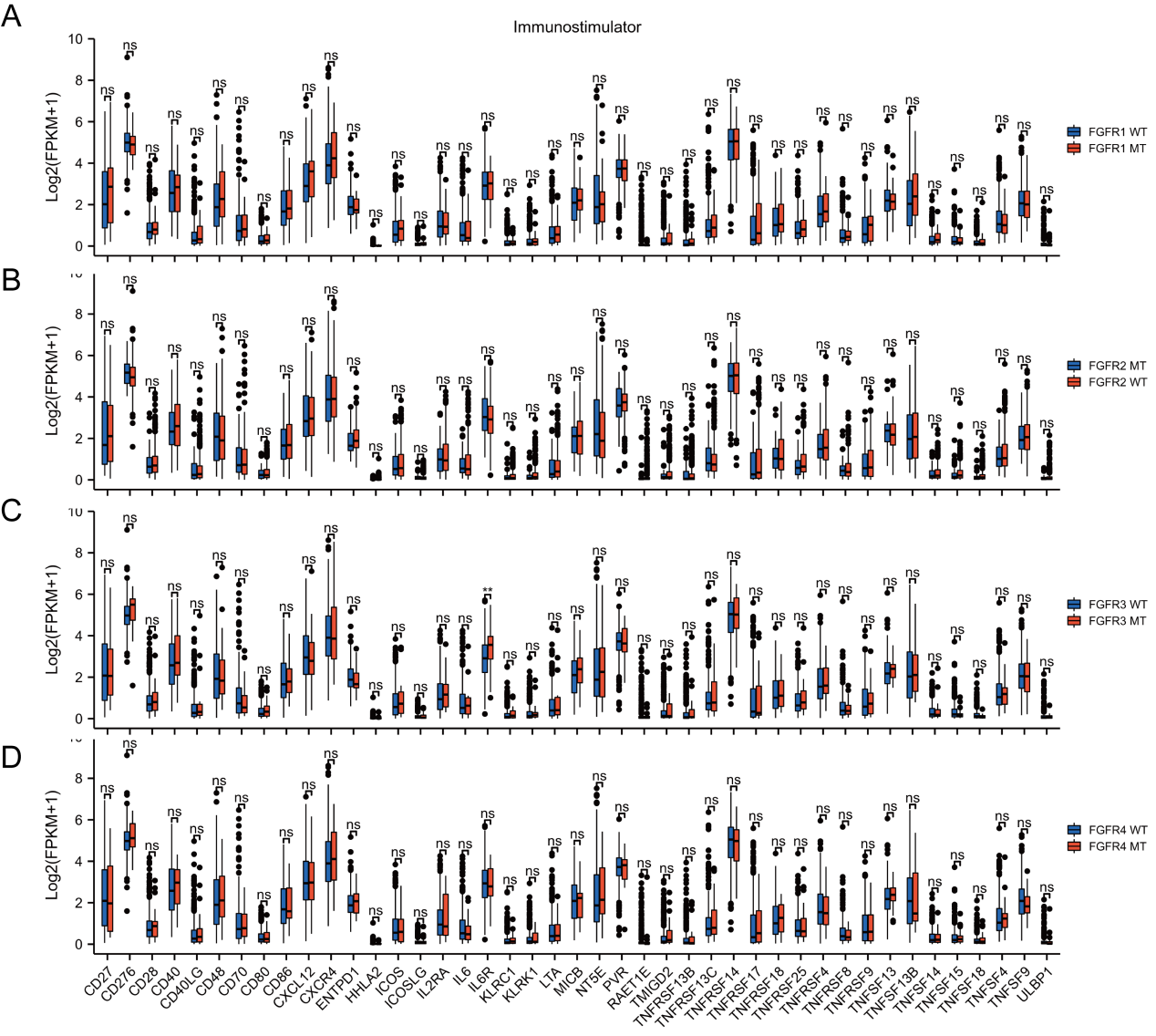


**FIGURE S7|** Comparison of expression of immuno-stimulator related genes in melanoma harboring FGFR mutation subtypes with their wild-type counterparts.


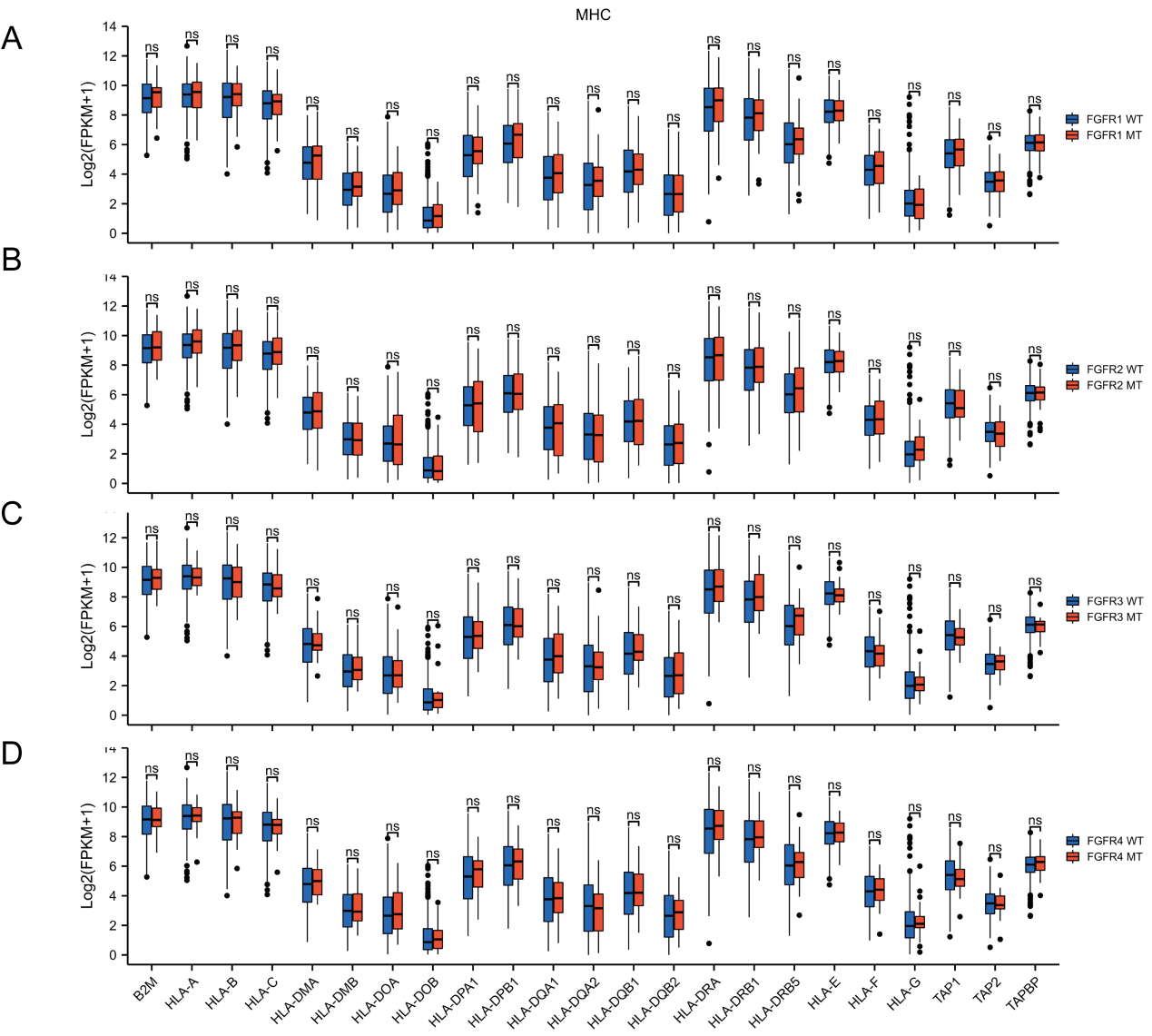


**FIGURE S8**| Comparison of expression of MHC molecule related genes in melanoma harboring FGFR mutation subtypes with their wild-type counterparts.


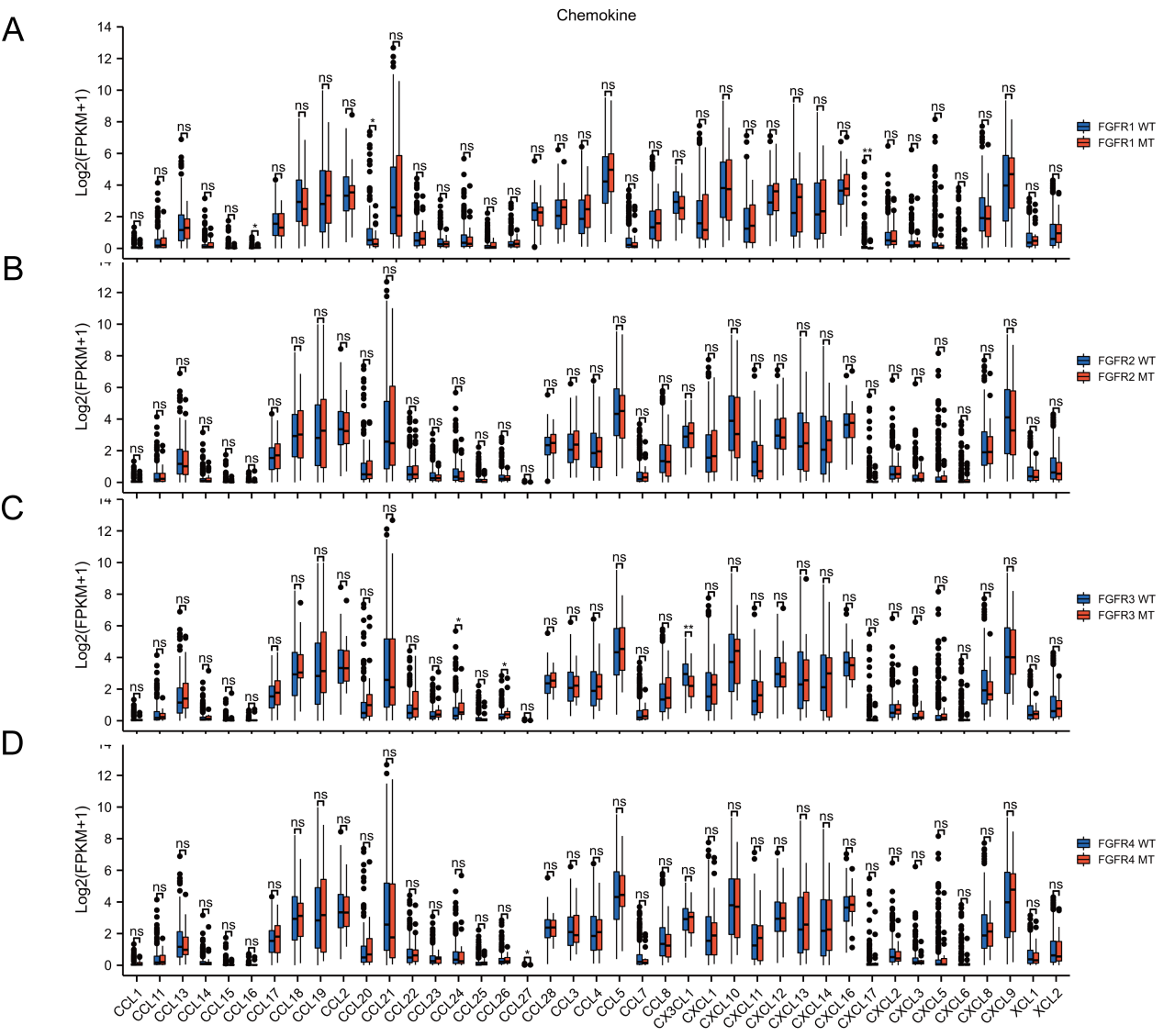


**FIGURE S9|** Comparison of expression of chemokines in melanoma harboring FGFR mutation subtypes with their wild-type counterparts.


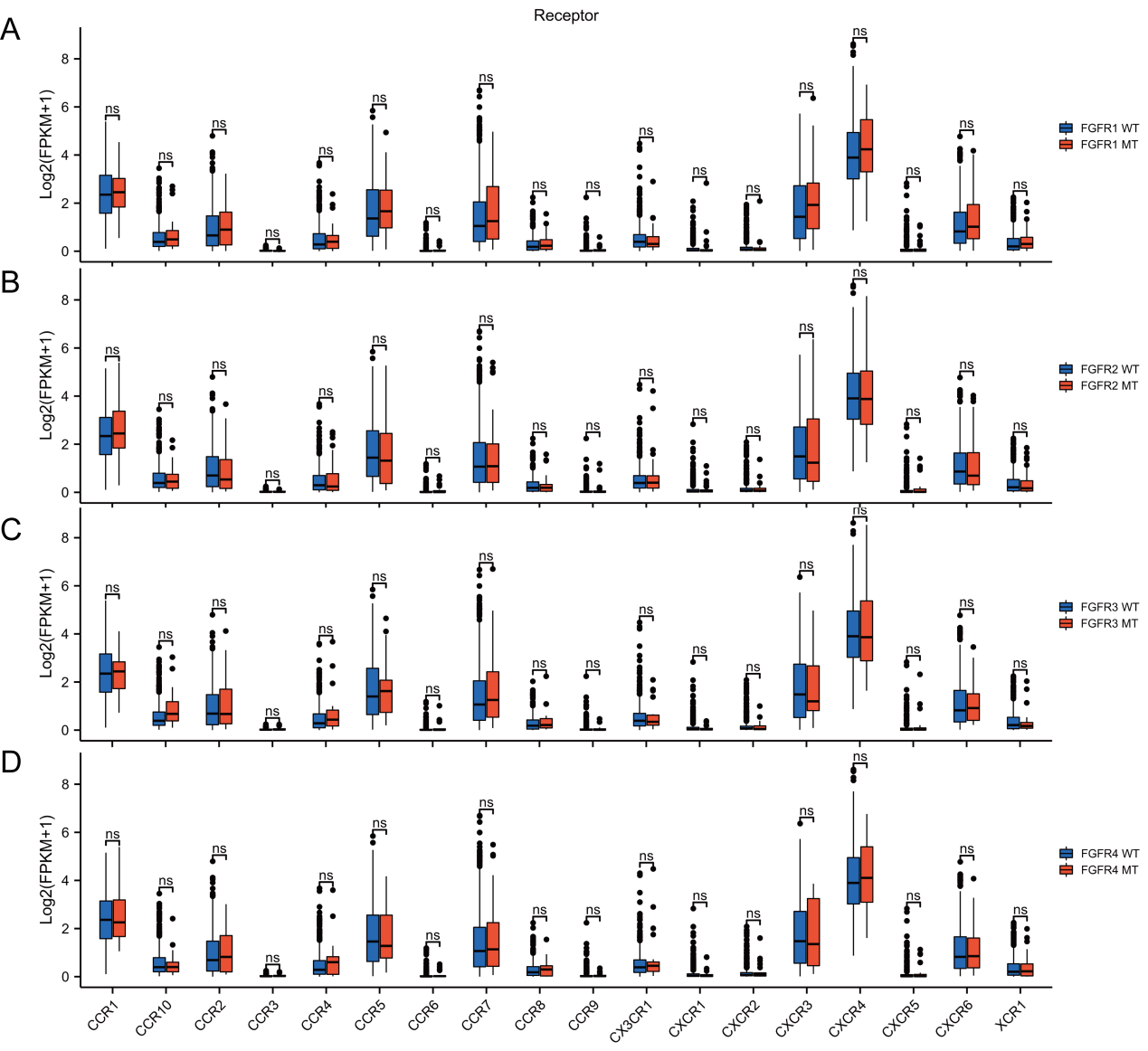


**FIGURE S10|** Comparison of expression of receptors in melanoma harboring FGFR mutation subtypes with their wild-type counterparts.


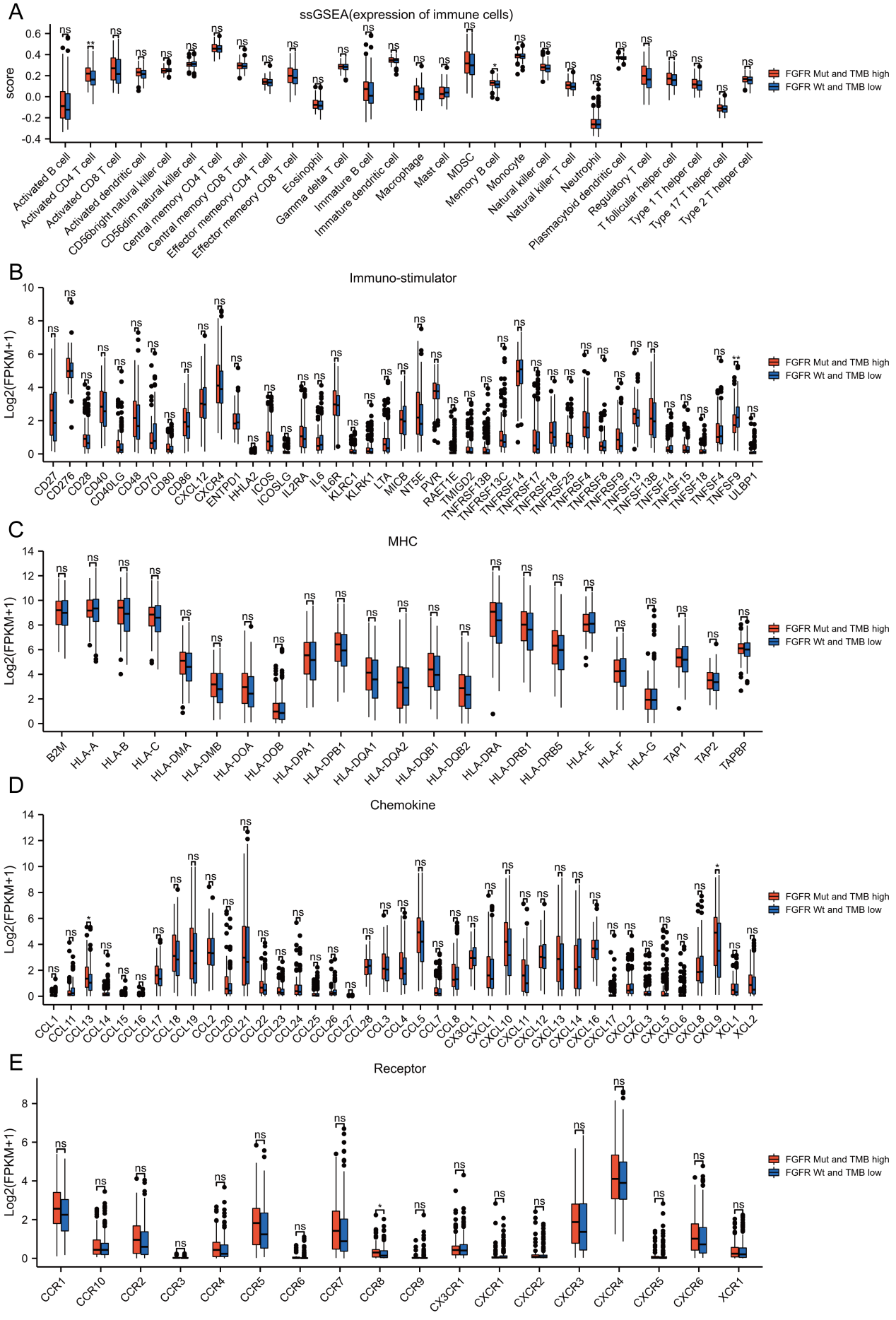


**FIGURE S11| (A)** Comparison of expression of immune cells between melanoma patients with FGFR^Mut^TMB^high^ and those with FGFR^Wt^TMB^low^. (**B**) Comparison of expression of immune-stimulators between melanoma patients with FGFR^Mut^TMB^high^ and those with FGFR^Wt^TMB^low^. (**C**) Comparison of expression of MHC between melanoma patients with FGFR^Mut^TMB^high^ and those with FGFR^Wt^TMB^low^. (**D**) Comparison of expression of chemokines between melanoma patients with FGFR^Mut^TMB^high^ and those with FGFR^Wt^TMB^low^. (**E**) Comparison of expression of receptors between melanoma patients with FGFR^Mut^TMB^high^ and those with FGFR^Wt^TMB^low^.
